# Blocking cholesterol storage to treat Alzheimer’s disease

**DOI:** 10.37349/ent.2021.00014

**Published:** 2021-12-30

**Authors:** Ta Yuan Chang, Catherine C. Y. Chang, Taylor C. Harned, Adrianna L. De La Torre, Junghoon Lee, Thao N. Huynh, James G. Gow

**Affiliations:** Department of Biochemistry and Cell Biology, Geisel School of Medicine at Dartmouth, Hanover, NH 03755, USA

**Keywords:** Alzheimer’s disease, cholesterol acyltransferase, cholesterol

## Abstract

Cholesterol serves as an essential lipid molecule in various membrane organelles of mammalian cells. The metabolites of cholesterol also play important functions. Acyl-coenzyme A: cholesterol acyltransferase 1 (ACAT1), also named as sterol *O*-acyltransferase 1, is a membrane-bound enzyme residing at the endoplasmic reticulum (ER). It converts cholesterol to cholesteryl esters (CEs) for storage, and is expressed in all cells. CEs cannot partition in membranes; they can only coalesce as cytosolic lipid droplets. Excess CEs are found in the vulnerable region of the brains of patients with late-onset Alzheimer’s disease (AD), and in cell and mouse models for AD. Reducing CE contents by genetic inactivation of *ACAT1*, or by pharmacological inhibition of ACAT is shown to reduce amyloidopathy and other hallmarks for AD. To account for the various beneficial actions of the ACAT1 blockade (A1B), a working hypothesis is proposed here: the increase in CE contents observed in the AD brain is caused by damages of cholesterol-rich lipid rafts that are known to occur in neurons affected by AD. These damages cause cholesterol to release from lipid rafts and move to the ER where it will be converted to CEs by ACAT1. In addition, the increase in CE contents may also be caused by overloading with cholesterol-rich substances, or through activation of *ACAT1* gene expression by various proinflammatory agents. Both scenarios may occur in microglia of the chronically inflamed brain. A1B ameliorates AD by diverting the cholesterol pool destined for CE biosynthesis such that it can be utilized more efficiently to repair membrane damage in various organelles, and to exert regulatory actions more effectively to defend against AD. To test the validity of the A1B hypothesis in cell culture and *in vivo*, the current status of various anti-ACAT1 agents that could be further developed is briefly discussed.

## Introduction

Alzheimer’s disease (AD) is the most prevalent neurodegenerative disease. Currently, it has no cure. Accumulations of extracellular amyloid plaques (known as amyloidopathy) and intracellular neurofibrillary tangles (known as tauopathy) are hallmarks of AD. In addition, an increase in cholesteryl ester (CE) contents occurs in the vulnerable region of the late-onset AD (LOAD) brain. Cholesterol plays a vital role in the brain, with cholesteryl ester being storage form of cholesterol. CE cannot substitute the function of cholesterol. Biosynthesis of CEs is carried out by the enzymes acyl-coenzyme A: cholesterol acyltransferase 1 (ACAT1) and ACAT2 [named as sterol *O*-acyltransferase 1 (SOAT1) and SOAT2 in GenBank]. In most cells of the human body, including the cells of central nervous system (CNS), ACAT1 is the major isoenzyme. Preclinical evidence from several laboratories implicates ACAT1 as a molecular target for the treatment of AD. Here is a brief review of the current status of the ACAT1/AD research field and the small molecule ACAT inhibitors.

## Cellular cholesterol trafficking pathway in a single mammalian cell

**Figure 1.**
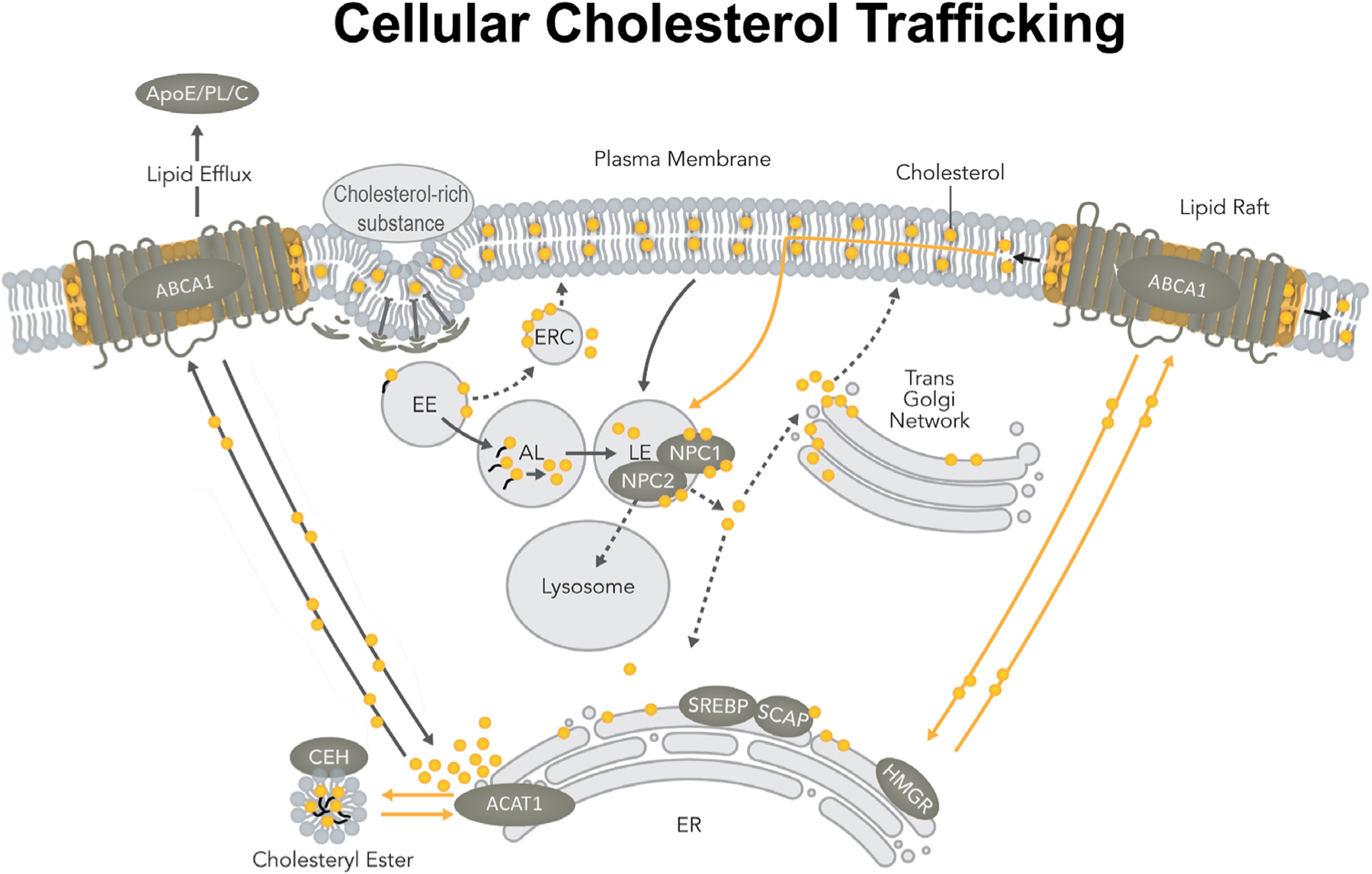
The cellular cholesterol trafficking pathway in a mammalian cell. ABCA1: ATP-binding cassette transporter 1; ApoE: apolipoprotein E; AL: acid lipase; C: cholesterol; CEH: cholesterol ester hydrolase; EE: early endosomes; ERC: endocytic recycling compartment; HMGR: HMG-CoA reductase receptors; LE: late endosomes; NPC: Niemann-pick type C proteins; PL: phospholipid; SREBP: sterol-regulatory-element binding protein; SCAP: SREBP cleavage activating protein *Note*. Adapted from “Cellular cholesterol homeostasis and Alzheimer’s disease,” by Chang TY, Yamauchi Y, Hasan MT, Chang C. J Lipid Res. 2017;58:2239–54 (https://doi.org/10.1194/jlr.R075630). © 2017 ASBMB. CC BY.

Cholesterol is a crucial lipid molecule that plays important roles in various membrane organelles, including the plasma membrane (PM), Golgi, endosomes, peroxisomes, mitochondria, and endoplasmic reticulum (ER). Cholesterol moves in and out of these membrane organelles dynamically. The transport and movement of cholesterol occur by at least three distinct trafficking pathways (Figure 1) [1].

The first pathway involves the endocytosis/phagocytosis of extracellular materials rich in cholesterol. These materials include cholesterol-rich lipoproteins such as low-density lipoproteins (LDL), lipidated ApoE, apoptotic cell debris, and damaged cell membranes. Upon binding to various cell surface receptors, these cholesterol-rich materials are internalized and enter the early endosome. CEs contained in materials, such as LDL, are cleaved in the early endosome by acid lipase, producing free cholesterol and fatty acids [2]. Next, cholesterol moves from the early endosome to the late endosome/lysosome. There, cholesterol interacts with NPC1 and NPC2; together, they work in concert to transport cholesterol out of the late endosomes/lysosomes [2, 3]. Organelles that receive cholesterol from NPC1/NPC2 may include PM, ER, as well as trans-Golgi network [4, 5].

The second pathway involves the transport of newly synthesized sterols from the site of sterol biosynthesis (ER) to the PM; this pathway is independent of NPC1 [1, 6]. At the PM, the microdomain enriched in the lipid efflux protein ABCA1 is a favorable recipient for these newly synthesized sterols, especially lanosterol [7]. Cholesterol from exogenous uptake and cholesterol produced from endogenous biosynthesis traverse through various membrane organelles, with these two pools merging at the PM.

The third pathway is comprised of the retrograde movement of the recycling cholesterol from the PM to the ER. This pathway is independent of NPC [8, 9]. At the PM, at least three different cholesterol pools are present [10]. Several proteins including the steroidogenic acute regulatory protein D4 [11, 12] and aster proteins/GRAM domain-containing proteins 1 [13, 14] are involved in the retrograde transport of cholesterol from PM to ER by non-vesicular lipid trafficking mechanism, perhaps through various PM/ER membrane contact site(s). In addition, ABCA1 is also involved in retrograde cholesterol transport, perhaps through a clathrin-independent endocytosis pathway [9]. These results together suggest that the movements of different cholesterol pools at the PM and the ER may be governed by different mechanisms.

## AD as a special lipid disease

AD is the most prevalent neurodegenerative disease. It is classified into either early-onset AD or LOAD, with 99% of the cases being LOAD. AD pathological hallmarks consist of extracellular amyloid plaques composed of amyloid beta peptides (Aβ) especially Aβ1–42, neurofibrillary tangles composed of misfolded and hyperphosphorylated tau, and chronic neuroinflammation [15]. In addition to amyloidopathy and tauopathy, a significant amount of lipid granules, perhaps as neutral lipids (i.e., triacylglycerols and CEs), also accumulate within the glia of the AD patient brain [16]. LOAD involves numerous environmental and genetic risk factors, with aging being the strongest-known risk factor. The major genetic risk factor for LOAD is the *ε4* allele of *ApoE* [17]. ApoE is the major lipid transport lipoprotein in the CNS. In addition to *APOE ε4*, about a dozen other genetic risk factors have been identified, including clustrin, ATP-binding cassette transporter A7, triggering receptor expressed on myeloid cells 2 (*TREM-2*), and *ABCA1*, all of which are involved in lipid metabolism [17–19]. Given the strong genetic association between LOAD and lipid metabolism, LOAD can be considered a unique lipid disease. Recent evidence from several laboratories supports this concept [20–22].

## CE and ACAT/SOAT

CE is the storage form of cholesterol. Normally, brain CE levels are less than 1% of total cholesterol content. However, in brain samples from LOAD patients, CE levels are increased by 1.8-fold in the vulnerable regions [23]. Similarly, in the vulnerable brain regions including entorhinal cortex of three different AD mouse models, the CE levels were 3- to 11-fold higher than those in control mice [23, 24]. At the cellular level, as shown in AD patient-derived neurons, increased CE contents are linked with tau pathology [25]. These findings indicate that CE content positively correlates with AD development. What causes CEs to be elevated in AD brains is still an active area of research.

**Figure 2.**
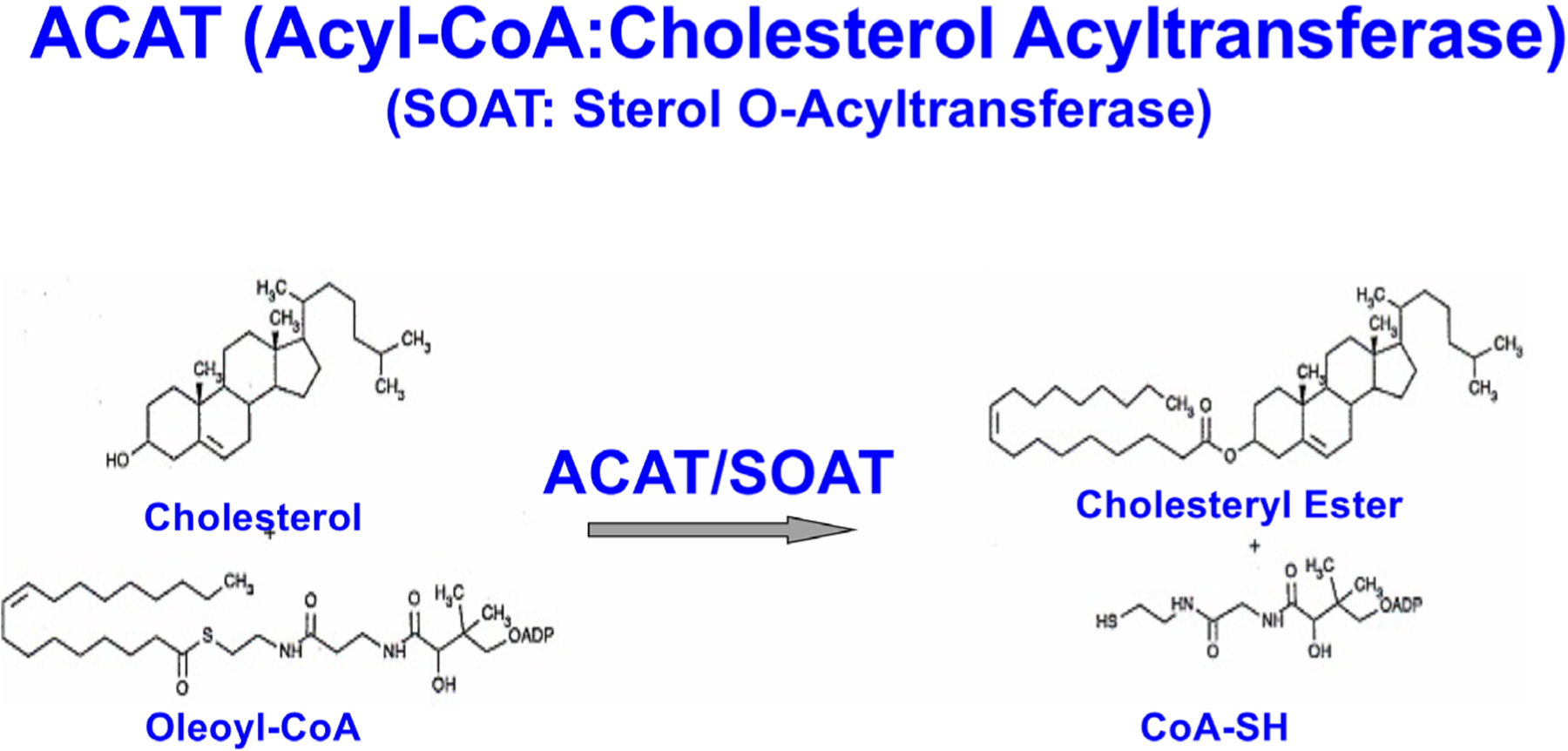
Biochemical reaction catalyzed by the enzyme ACAT/SOAT

CEs stored as cytoplasmic lipid droplets exist in a dynamic state. Cholesterol esterification by the ACAT1/SOAT1 and subsequent hydrolysis by neutral CE hydrolases constitute the cholesterol/CE cycle, thus allowing CEs to undergo continuous turnover [26]. There are two *ACAT* genes, *SOAT1* and *SOAT2*, encoding two homologous but distinct enzymes, ACAT1 [27] and ACAT2 [28–30], respectively. Both enzymes use long-chain fatty acyl-coenzyme A and sterols with 3-beta-OH at the steroid ring A position as substrates, this includes cholesterol and various oxysterols (Figure 2) [31]. ACAT1 is ubiquitously expressed in essentially all cell types, including in the brain. ACAT2 is mostly expressed in intestinal enterocytes and in hepatocytes, but it is also detectable in various peripheral tissues. ACAT1 and ACAT2 are integral membrane proteins located at the ER. Unlike many enzymes in the cholesterol biosynthesis pathway (such as 3-hydroxy-3-methylglutaryl-CoA reductase), neither ACAT1 nor ACAT2 is regulated by the transcription factor sterol response element binding protein 2 [32, 33]. Instead, both ACATs are allosterically activated by its substrate cholesterol or oxysterols [34–36].

## ACAT1 blockade and AD

Evidence from several laboratories at the preclinical level implicates ACAT1 as an important molecular target for the treatment of AD [37–43]. Mechanistically, ACAT1 blockade (A1B) offers multiple benefits to AD including the following: (1) *ACAT1* gene ablation increases the content of the neuroprotective oxysterol 24(*S*)-hydroxycholesterol in the AD mouse brain [39] and in the AD patient induced pluripotent stem cell derived human neuronal cells [25]. (2) In mouse and cell models for amyloidopathy and tauopathy, A1B increases autophagy and lysosomal biogenesis, which leads to the clearance of Aβ oligomers in microglia [41]; A1B also increases the clearance of misfolded tau in neurons [43, 44]. (3) In AD patient-derived neurons, CEs inhibits tau proteostasis through an unknown mechanism but is independent of amyloid precursor protein and Aβ. A1B is shown to reduce CE content which prevents the inhibitory effect of CEs on tau proteostasis [25]. (4) A1B decreases the protein content of mutant hAPP in an AD mouse model [39], and in AD patient induced pluripotent stem cell derived human neuronal cells [25]. (5) TREM-2 deficiency has been identified as a risk factor for LOAD. TREM-2 is a membrane receptor expressed in microglia, that mediates phagocytosis of cholesterol-rich myelin debris, as well as the subsequent clearance of building CEs from the cytosol. When microglia are exposed to myelin debris, the lack of TREM-2 causes these cells to accumulate large amounts of CEs; A1B has been shown to clear the CE buildup rapidly [43].

## Cholesterol loading, CE content, and the A1B hypothesis

Cholesterol, phosphatidylcholine, and sphingolipids can come together to form lipid raft domains/lipid clusters within cellular membranes. These lipid-rich rafts and raft-like domains are present in PM, Golgi, endosomes, and mitochondrial-associated membranes of the ER. In amyloidopathy, oligomeric Aβ interacts with cholesterol-rich lipid rafts; it is known that these interactions cause damage to the lipid rafts [1]. Here we posit that the membrane damages generated by oligomeric Aβ cause a portion of cholesterols to dissociate from the lipid rafts and move to the ER by default, followed by esterification by ACAT1. This hypothesis may explain why amyloidopathy leads to CE content to increase in various AD systems. In AD, the other major toxin is the misfolded tau. We speculate that misfolded tau may act in a similar manner as oligomeric Aβ, i.e., by damaging the lipid rafts thereby increasing the ER cholesterol content available to ACAT as substrate by default. This scenario may apply mainly to neurons that are affected by AD. A different scenario may account for the increases in CEs in microglia. Microglia are phagocytes and they actively phagocytose various substances rich in cholesterol. For examples, under normal conditions, microglia engulf axons and synapses to eliminate immature or less active inputs. In aging and/or in various disease conditions, myelin debris and other cholesterol-rich substances, such as cell debris, apoptotic neurons, as well as damaged membrane organelles, may accumulate in the brain [45]. A special population of microglia designated as TREM-2 positive microglia actively phagocytose these materials [46]. These cholesterol-rich substances may also provide a rich source of cholesterol as substrate for ACAT1. In the mouse cortex, the *ACAT1* transcript in microglia is significantly higher than those in other cell types in the CNS [47]. Based on these analyses, we predict that the CE content in microglia may be higher than those in other cell types, perhaps in brain region specific manner. The scenario discussed above focuses on increasing the cholesterol supply to ACAT as a substrate as the cause for the increases in CEs. In addition to an increase in substrate supply, cell culture study in macrophages showed that *ACAT1* gene and ACAT1 protein content are up-regulated by the proinflammatory cytokine tumor necrosis factor-alpha [48]. Since AD involves chronic inflammation, it is possible that brain inflammation in AD leads to the accumulation of several proinflammatory cytokines which then cause the increase in *ACAT1* gene expression and in ACAT1 protein content. The increased ACAT1 protein content will lead to increased CE production.

To provide a rationale for the A1B effects in a single cell, we posit that A1B causes the cholesterol pool at the vicinity of ACAT1 to build up. Cholesterol in excess of binding to PC/sphingolipid becomes “mobile cholesterol”, which tends to move away from the ER to other membrane organelles, including the Golgi, endosome, PM, etc., thus increasing their respective free cholesterol contents [49]. In AD, the mobile cholesterol produced by A1B helps to repair the functional deficiency of lipid rafts in the membranes damaged by oligomeric Aβ and/or by tau. A1B also increases regulatory responses located at the ER which facilitate cellular cholesterol homeostasis [1]. Targeting microglia has been considered as an effective approach to treat AD and other neurodegenerative diseases. It will be interesting to test whether the effects of A1B are more robust in microglia, especially when they are cholesterol-loaded.

## The pros and cons of the A1B approach to treat LOAD

The validity of the A1B hypothesis needs to be further tested in various cell culture systems and *in vivo*. Aging and ApoE4 are two major risk factors for LOAD. Therefore, the effects of A1B need to be examined in animal models for aging, preferably in ApoE isoform-specific manner. The excess build-up of free cholesterol in cells can cause toxicity. To prevent this toxicity, the major lipid efflux protein ABCA1 plays a key role in assisting the disposal of cholesterol at the PM [50]. Fortunately, in various cell types examined, A1B increased the protein content of ABCA1, as well as the capacity of ABCA1 to efflux cellular cholesterol [51]. Therefore, in normal situation, A1B is not expected to cause free cholesterol to build up intracellularly. Still, it is important to test the effects of A1B in cells without significant deficiency in ABCA1 and/or in other related proteins involved in the reverse cholesterol pathway. It is also important to know that in mouse models, genetic inactivation of *ACAT1* caused dry eye syndrome, hair loss, and increased leukocyte production. In our opinion, the adverse effects can be used to predict drug overdose of A1B *in vivo*.

## Possible link between the *ACAT1/SOAT1* single-nucleotide polymorphism with AD risk

A recent study reported that among one of the four common *ACAT1*/*SOAT1* polymorphisms investigated, one protective haplotype and one risk haplotype for the development of dementia have been found [52]. The first single-nucleotide polymorphism (SNP) is located in *exon 14* [53], but the change in nucleotide does not cause change in amino acid (i.e., a synonymous mutation). The second SNP is located at the 3’ UTR region of the *ACAT1*/*SOAT1* mRNA [27], whose link with AD risk has been previously reported [54]. The functional significance of these two SNPs is unknown at present and awaits further investigation.

## Small molecule ACAT inhibitors to treat atherosclerosis or AD

ACAT has been a drug target to treat atherosclerosis and many ACAT inhibitors have already been produced. The characteristics of several ACAT inhibitors and their status as anti-atherosclerosis drugs are listed (Table 1).

CI1011 is an isoform non-specific ACAT inhibitor; it inhibits both ACAT1 and ACAT2 with modest inhibition constant (Ki). CI1011 successfully completed clinical testing, however, it was abandoned because it lacked efficacy as a supplement to the statin drugs (the 3-hydroxy-3-methylglutaryl-coenzyme A reductase inhibitors), to further reduce serum cholesterol levels in patients with hypercholesterolemia [55]. The lack of efficacy of CI1011 was in part due to its drug-drug interaction with statins [56].

Pactimibe is a second-generation derivative of CI1011; it also failed to supplement the actions of statins in patients [57].

**Figure 3.**
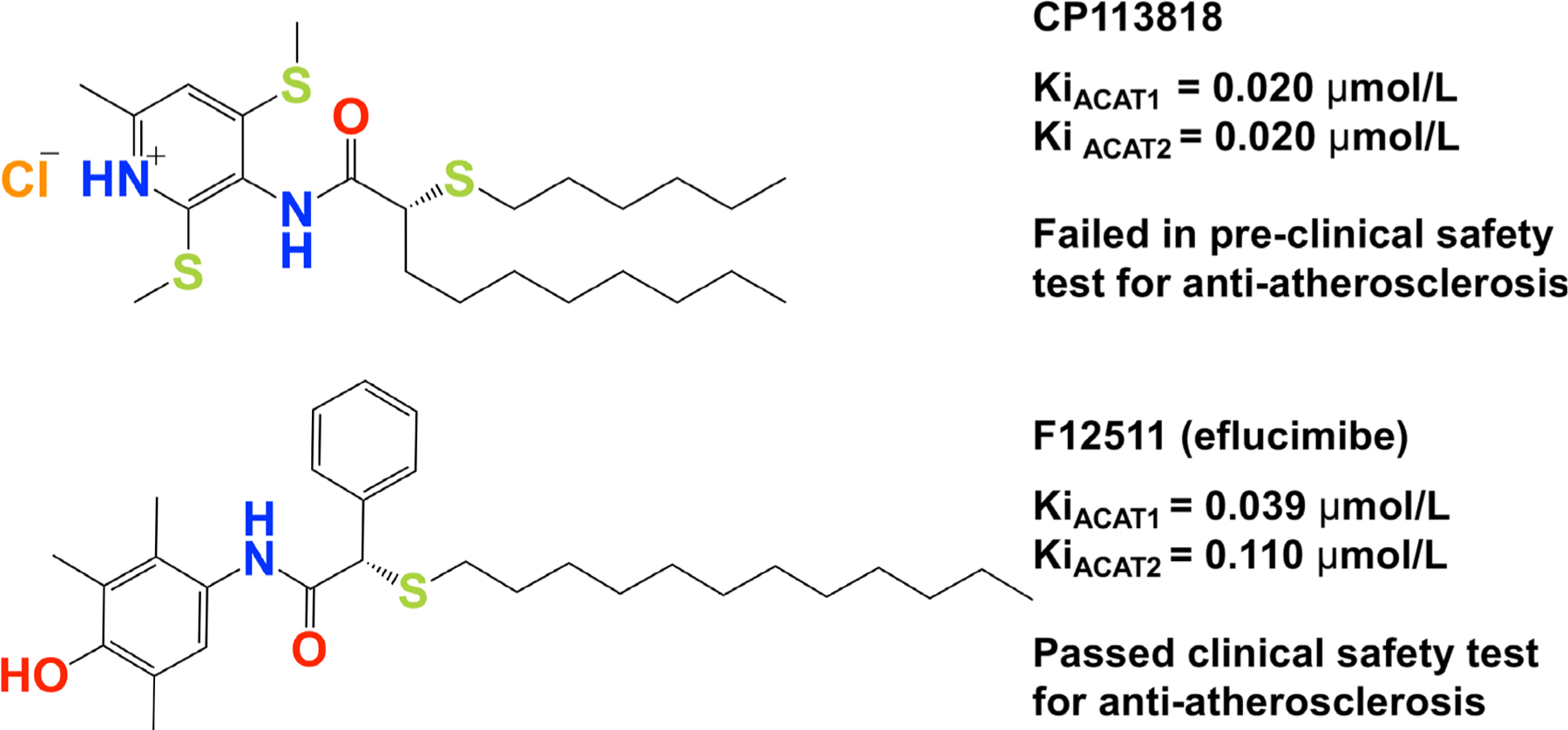
Comparison of 2 high-affinity ACAT inhibitors CP113818 and F12511

CP113818 is a high-affinity, isoform-non-specific ACAT inhibitor (Ki = 0.02 μmol/L). Animal studies showed that CP113818 accumulated in adrenal cell membranes and caused toxicity (unpublished observations made by scientists at Pfizer). CP113818 possesses an asymmetric carbon (Figure 3). This asymmetry is needed in order for CP113818 to act as a potent ACAT inhibitor. The enantiomeric isomers of CP113818 (and its close analogs) are inactive as ACAT inhibitors, however, they also cause severe adrenal toxicities [58]. Thus, the toxicity of CP113818 (and their close analogs) might be caused by their properties as membrane-active compounds, which enabled them to be highly enriched in adrenal cell membranes, instead of its ability to inhibit ACAT [59]. Supporting this interpretation is the genetic evidence that the adrenal functions of mice globally devoid of *ACAT1* is normal [60].

To improve the pharmacological properties of CP113818, scientists at Pierre Fabre in France produced F12511 as a second-generation derivative of CP113818. F12511 also contains an asymmetric center (Figure 3) and is a high-affinity ACAT1 inhibitor (Ki = 0.039 μmol/L) that also inhibits ACAT2 with Ki = 0.11 μmol/L. Unlike 113818, F12511 did not cause adrenal toxicity in animals, and passed the clinical safety test in humans as an anti-atherosclerosis drug candidate but F12511 was abandoned for undisclosed reason(s) [61, 62].

K604 is a second-generation derivative of pactimibe (described earlier) and is a high-affinity ACAT1-specific inhibitor with Ki = 0.45 μmol/L [63]. It also passed the clinical safety test but was abandoned for undisclosed reason(s).

The analysis described in Figure 2 shows that the toxicity of the ACAT inhibitors can be avoided or minimized by careful chemical modification of the lead compound(s). In a mouse model for AD, Kovacs and colleagues showed that, when delivered by implanting CP113818 as a slow releasing pellet underneath the mouse skin, CP113818 was effective in suppressing amyloidopathy [38]. This was the first animal study suggesting that certain ACAT inhibitors originally designed for treating atherosclerosis can be repurposed to treat AD. Whether F12511 or K604 is effective in mouse models for AD has not been reported in the literature.

## Adeno-associated viral expression of small interfering RNA against *Acat1*/*Soat1* to treat AD

Adeno-associated viruses (AAVs) are considered as major gene therapy vectors because they produce long-term (at least one year), stable, episomal expression of the viral transgene, provoke only a minimal inflammatory response, have minimal oncogenic potential, and can infect and transduce various postmitotic cells including neurons and astrocytes. Many laboratories have used this strategy as potential gene therapy to treat animal models for neurodegenerative diseases [64]. In a mouse disease model for early-onset AD, delivering a specific AAV-siRNA against *Acat1* by direct injection into mouse hippocampus significantly reduced ACAT1 enzyme activity in the brain and reduced amyloidopathy [40]. This work suggests that AAV-siRNA against *Acat1* can be considered as a potential therapy to treat AD and other related neurodegenerative diseases.

## Conclusion and perspectives

AD is a neurodegenerative disease that currently has no cure. LOAD involves numerous environmental and genetic risk factors, and leads to cholesterol dyshomeostasis in the CNS. In various experimental AD systems, A1B has been reported to provide multiple benefits. Here we propose a simple working hypothesis to account for the beneficial actions of A1B within a single cell:

- In neurons, Aβ and misfolded tau dissociate cholesterol from the lipid clusters in various membrane organelles, causing more cholesterol to move to the ER by default and to serve as ACAT1 substrate.
- In microglia, aging and other AD risk factors ultimately increase cholesterol supply to ACAT1.
- In various cell types of the brain affected by AD, TNF-alpha and other proinflammatory factors increase *ACAT1* gene expression and ACAT1 protein content.
- In various cell types, blocking ACAT1 causes the cholesterol substrate pool to move away from the ACAT1 domain at the ER, and into various other membrane organelles, including the Golgi, endosome, PM, etc., where it will be utilized more efficiently.

The validity of this hypothesis needs rigorous testing in animal studies and in human systems. The current status of various small molecule ACAT inhibitors to treat atherosclerosis and AD, and the use of AAV-siRNA against *Acat1* to treat mouse model of AD is reviewed here. Hopefully, these agents will be used to test this hypothesis, and to be further developed to help ameliorate LOAD. Other neurodegenerative diseases, including Niemann-pick type C disease [65, 66], amyotrophic lateral sclerosis [67], Huntington disease [68, 69], and adult glioma [70, 71], also involve cholesterol dyshomeostasis in the brain. Diverting cholesterol from conversion to CEs by inhibiting the cholesterol storage enzyme ACAT1 to increase cholesterol utilization may also benefit these diseases.

## Figures and Tables

**Table 1.** ACAT inhibitors for anti-atherosclerosis purpose

Inhibitors	Ki (μmol/L)		Status
	ACAT1	ACAT2	
CI976	5.9	2.1	Failed at pre-clinical stage (Parke-Davis)
ATR101 (nevanimibe)	0.23		Failed at pre-clinical stage (Parke-Davis)
CP113818	0.02	0.02	Failed at pre-clinical stage (Pfizer)
CI1011	19	19	Abandoned after phase 3 (Pfizer)
CS505 (pactimibe)	4.9	3	Abandoned after phase 3 (Sankyo)
K604	0.45	102.9	Abandoned after phase 2 trial (Kowa)
F12511	0.039	0.11	Abandoned after phase 1 trial (Lilly)

## Abbreviations

Aβ: amyloid beta peptides
A1B: acyl-coenzyme A: cholesterol acyltransferase 1 blockade blockade
AAVs: adeno-associated viruses
ABCA1: ATP-binding cassette transporter A1
ACAT1: acyl-coenzyme A: cholesterol acyltransferase 1
AD: Alzheimer’s disease
ApoE: apolipoprotein E
CEs: cholesteryl esters
CNS: central nervous system
ER: endoplasmic reticulum
Ki: inhibition constant
LOAD: late-onset of Alzheimer’s disease
NPC: Niemann-pick type C proteins
PM: plasma membrane
SNP: single-nucleotide polymorphism
SOAT1: sterol *O*-acyltransferase 1
TREM-2: triggering receptor expressed on myeloid cells 2
